# Impact of electronic patient-reported outcomes (ePRO) presentation in pancreatic cancer tumor board discussions on cancer outcomes: the INSPIRE intervention

**DOI:** 10.1186/s12885-025-14847-w

**Published:** 2025-12-30

**Authors:** Garrett Bourne, Nicole Henderson, Joud El Dick, Luqin Deng, Jeffrey Franks, Courtney P. Williams, Cameron Pywell, J. Bart Rose, Grant R. Williams, S. M. Qasim Hussaini, Ryan D. Nipp, Gabrielle Rocque

**Affiliations:** 1 Division of hematology and oncology, O’Neal Comprehensive Cancer Center at UAB, Birmingham, AL 35294 USA; 2 Division of General Internal Medicine & Population Science, Department of Medicine at UAB, Birmingham, AL 35294 USA; 3 O’Neal Comprehensive Cancer Center, University of Alabama, Birmingham, USA; 4 OU Health Stephenson Cancer Center, Oklahoma City, OK 73104 USA

**Keywords:** Pancreatic ductal adenocarcinoma, Geriatric assessment, Electronic patient-reported outcomes, Multidisciplinary tumor boards

## Abstract

**Background:**

Pancreatic ductal adenocarcinoma (PDAC) primarily affects older adults and has a poor prognosis. Although tools like geriatric assessments and electronic patient-reported outcomes (ePRO) can guide treatment, they are underutilized in clinical practice. This secondary analysis of the INSPIRE pilot intervention, a pilot intervention that assessed the utility of incorporating data on patient preferences and frailty into multidisciplinary tumor board (MDTB) discussions, evaluated the clinical impact of incorporating patient preferences and frailty data into MDTB discussions.

**Methods:**

The study included patients aged ≥ 60 years with PDAC enrolled in the INSPIRE intervention at the University of Alabama at Birmingham. Patients discussed at MDTBs with adequate medical records who did not forgo treatment initially were included. A control group comprised patients who completed preference and frailty surveys but whose ePRO data were not presented at MDTBs. Outcomes analyzed included treatment consistency with preferred National Comprehensive Cancer Network (NCCN) regimens based on fitness, unplanned treatment modifications, and healthcare utilization within six months of treatment initiation. Data were extracted from medical records and statistical analysis employed log-rank tests from cumulative incidence functions.

**Results:**

Among 121 patients (24 intervention, 97 controls), the median age was 70 years. Compared to controls, the intervention group had fewer comorbidities (8% vs. 25% with no comorbidities, V = 0.22), a higher proportion of non-White patients (42% vs. 25%, V = 0.15), more resectable disease (48% vs. 35%, V = 0.14), and higher frailty rates (42% vs. 31%, V = 0.11). Intervention patients showed slightly higher consistency with NCCN preferred regimens based on fitness (63% vs. 60%, V = 0.02), fewer unplanned treatment modifications (54% vs. 68%, V = 0.12), and lower hospital admissions (33% vs. 50%, V = 0.13).

**Conclusion:**

The INSPIRE intervention demonstrated promising signals when aligning treatment regimens with patient capabilities and preferences, potentially reducing unplanned treatment modifications and hospital admissions. Larger studies are needed to confirm these exploratory results and assess broader applicability.

**Supplementary Information:**

The online version contains supplementary material available at 10.1186/s12885-025-14847-w.

## Background

Pancreatic ductal adenocarcinoma (PDAC) is a cancer primarily affecting older adults, with a median age at diagnosis of 71 years [1]. It is often detected late in the disease course, with approximately 50% of patients presenting with stage IV disease [2]. Many PDAC therapies are toxic and invasive and, for those eligible to receive them, patients often receive intense combination chemotherapy regimens, such as FOLFIRINOX (5-floururacil, irinotecan, and oxaliplatin) in addition to complex surgery, including a Whipple procedure that removes the pancreas and sections of the liver and small bowel. As such, PDAC treatment decision-making is heavily dependent on patient fitness to estimate treatment tolerability. The overall prognosis for the majority of patients diagnosed with PDAC is poor, with estimates suggesting a 5-year survival of only 10%.^2^ In light of this prognosis, many patients with PDAC prefer treatment approaches that maximize health-related quality of life (HRQOL) over quantity of life [3]. Some commonly reported preferences for patients with cancer, including several PDAC studies, include: maintenance of cognition, functional ability and quality of life, out-of-pocket expenses, and reducing treatment burdens on family and friends [4–7]. However, data indicates that only 37% of patients with cancer report that their preferences regarding cancer therapy were evaluated prior to treatment initiation highlighting an area for improvement [3]. 

A key consideration for decision-making in cancer care is frailty and treatment tolerability. Accurately assessing a patient’s fitness and/or frailty is a strong predictor of their capacity to tolerate cancer therapy [8]. Oncologists’ ability to accurately and/or completely assess patients’ frailty status frequently relies on subjective assessments [9]. These performance status assessments often have both considerable inter-clinician variability and patient-physician variability [9–11]. However, systematic tools are available to enhance such assessments, especially for older patients. For example, the geriatric assessment (GA) represents a systematic evaluation for assessing a broad array of patient health domains (e.g. function, nutrition, cognition, comorbidity, social support etc.) [12]. Multiple randomized trials have demonstrated improved communication, reduced chemotherapy toxicity, and improved HRQOL when using the GA in patients with cancer compared to standard of care [13–17]. In gastrointestinal (GI) cancers specifically, an electronic version of the GA has been shown to aid in reliable predictions of survival and to help inform treatment decision-making [18]. Despite potential to improve decision-making, data indicates that only 20% of oncologists consistently use GA in routine practice [19]. Common barriers to use in routine cancer care include a lack of training/understanding about ePROs, as well as a lack of both time and support staff [20–22]. As such, incorporating detailed performance status assessments and patient treatment preferences into the medical decision-making process in a standardized and efficient manner remains a key challenge.

The **IN**tegrating **S**ystematic **P**at**I**ent-**R**eported **E**valuations (INSPIRE) pilot intervention assessed the utility of incorporating data on patient preferences and frailty into MDTB discussions [23]. We anticipated that presenting ePROs during the pancreatic cancer MDTB would enable a standardized setting in which this data could be succinctly presented and utilized for treatment-related decisions. In this secondary analysis of the pilot intervention, we hypothesized the INSPIRE intervention would improve treatment consistency with national guidelines by enabling more accurate patient fitness assessments, as well as improve key clinical outcomes, including reducing unplanned chemotherapy dose modifications, emergency department (ED) visits, and hospital admissions.

## Methods

### Study design and patient population

**Fig. 1:**
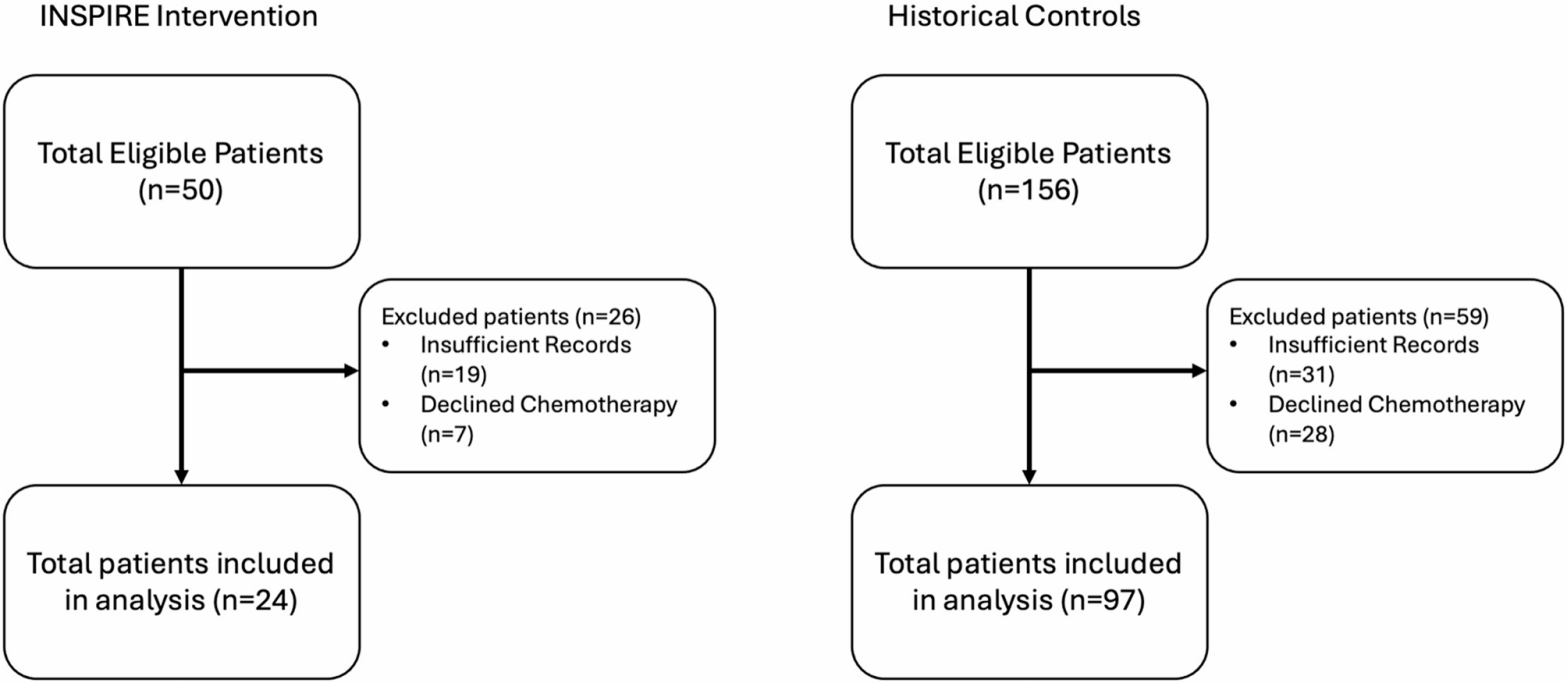
Consort diagram

This study is a secondary and exploratory analysis of clinical outcomes from a pilot feasibility trial conducted at the O’Neal Comprehensive Cancer Center at the University of Alabama at Birmingham (UAB) from September 2022 to June 2023. The pilot trial assessed both the feasibility of integrating ePROs into the pancreatic MDTB and the qualitative changes in discussion content resulting from this intervention [23]. Adult patients aged ≥ 60 years, with either a pathologic or clinical diagnosis of PDAC, and a plan to be discussed at pancreatic MDTB were eligible for this study. Any patients unable to read and/or speak English, provide informed consent, with life expectancy less than 3 months, or seen only for second opinion were excluded from the primary pilot study. Patients were further excluded from the secondary cohort study either due to medical records that didn’t contain sufficient data to be included in analysis or deciding to forgo treatment altogether upfront (Fig. 1). This study was approved by the UAB Institutional Review Board (IRB-300007868).

### Current institutional practice

At UAB, all patients with a new or suspected diagnosis of pancreatic cancer are presented at the weekly pancreatic MDTB, during which a range of specialists (medical oncologists, surgical oncologists, radiation oncologists, diagnostic and interventional radiologists, pathologists) convene to discuss each patient and formulate a multidisciplinary recommendation for cancer therapy. Since 2017, new patients at UAB aged ≥ 60 years with a diagnosis of a gastrointestinal malignancy seen in the medical oncology clinic are offered the opportunity to complete a modified version of the GA at the time of consultation [24]. The GA was later incorporated into an electronic, ePRO-capture survey tool entitled the **W**eb-**E**nabled Cancer and Aging Resilience Evaluation (WeCARE+). The tool allows for patient reporting of ePROs, including those assessing the presence of impairments in instrumental activities of daily living, recent falls, medical comorbidities, medications, psychological state, social support, cognition, nutritional status and patient preferences for treatment [18, 25]. However, the WeCARE + survey was not utilized in surgical clinics, its use by clinicians varied, and the results were not previously shared during MDTBs, which limited its reach.

### INSPIRE intervention

#### Intervention

 Prior to MDTB, all patients receiving the INSPIRE pilot intervention completed a baseline WeCARE + survey, as well as a survey asking them to rank order their top three concerns regarding available therapeutic options. A research coordinator then created a standardized, single-slide dashboard with patient-reported data derived from previously completed surveys for each intervention patient. These dashboards were then displayed to participants during pancreatic MDTBs prior to discussion on treatment recommendations. The tools included in the WeCARE + survey as well as an example MDTB patient dashboard can be seen in appendices A-C.

### Historical control

 Patients with PDAC in the pre-intervention period who completed the WeCARE + survey but whose data were not presented at MDTB were included in the analysis as controls.

### Outcomes

Outcomes evaluated in this secondary analysis were obtained via electronic medical record review by two independent chart abstracters (GB, JE) and included: (1) treatment consistency with National Comprehensive Cancer Network (NCCN) guidelines based on fitness level, (2) unplanned treatment modifications, and (3) healthcare utilization at 6-months post-treatment initiation.

#### Treatment consistency with national guidelines based on fitness

Consistency was defined by having an initial therapy that aligned with “preferred regimen” recommendations from the NCCN, according to degree of disease burden and patient performance status. The NCCN guidelines recommend more robust, intensive therapy for fit patients with resectable disease while recommending less intensive therapy for frail patients with more advanced disease [26]. NCCN guidelines utilize ECOG scores to incorporate patient performance status into their treatment regimen recommendations. We converted GA frailty scores into ECOG scores as follows: “frail” = ECOG 3, “pre-frail” = ECOG 2, “robust” = ECOG 1. Any patient receiving a treatment regimen other than an NCCN designated “preferred regimen” (including those receiving “other recommended regimens”) were categorized as not preferred. This includes individuals who received more intensive and less intensive treatments than were recommended by the preferred NCCN guidelines.

#### Unplanned treatment modifications

 Modifications were defined as chemotherapy dose reductions and/or delays within six months post-PDAC treatment initiation.

Healthcare utilization Healthcare utilization was defined as ED visits and hospital admissions within six months post-PDAC treatment initiation.

### Variables

Patient sociodemographics obtained via electronic health records included: age at diagnosis, sex, and race (White, Non-White [Black/African American, American Indian/Native Alaskan, Asian, or other/unknown]). Patients’ clinical characteristics, obtained via electronic health records and ePROs, included number of medical comorbidities (0, 1, 2, 3+) and GA-based frailty status (frail, pre-frail, robust). A classification of “pre-frail” indicates the patient met one or two of nine possible criteria for frailty and a classification of “robust” indicates they met no criteria for frailty. Tumor resectability obtained via electronic health records and was characterized as resectable, borderline resectable, or locally advanced/metastatic based on the NCCN guidelines.

### Statistical analysis

Summary statistics, including the median and interquartile range for continuous variables and frequencies and percentages for categorical variables, were calculated. Frequencies of sociodemographic characteristics, guideline consistent treatment, unplanned treatment modifications, and healthcare utilization were compared for INSPIRE intervention and control patients using Cramer’s V (categorical variables) or Cohen’s d (continuous variables) effect sizes. When comparing across two sample, a Cohen’s d of 0.2, 0.5, and 0.8 indicates a small, medium, and large effect, while a Cramer’s V of 0.1, 0.3, and 0.5 indicates a small, medium, and large effect, respectively [27, 28]. Time to first unplanned treatment modifications and healthcare utilization were compared for INSPIRE intervention and control patients using log-rank tests from cumulative incidence functions, with censoring occurring at 6 months post-initiation of chemotherapy. All analyses were computed using SAS Version 9.4.

## Results

### Sample characteristics

Of the 206 eligible patients for review (*n* = 50 INSPIRE, *n* = 156 historical control), 26 and 59 were excluded from the INSPIRE and historical control arms respectively, either due to medical records that didn’t contain sufficient data to be included in analysis or deciding to forgo treatment altogether upfront (Fig. 1). Demographics of patients excluded from this secondary analysis are included in Supplementary Table 1 and notably were often more frail and had more medical comorbidities than patients included in the analysis. Ultimately, 48% (*n* = 24) of intervention and 62% (*n* = 97) of historical control patients were included in this secondary analysis (*N* = 121). Compared to historical control patients, those in the INSPIRE intervention often had comorbid conditions (25% vs. 8% with no comorbidities, V = 0.22), more often were non-White (42% vs. 25%, V = 0.15), more often had resectable disease (48% vs. 35%, V = 0.14), and more often were frail (42% vs. 31%, V = 0.11; Table 1).

**Table 1:** Sample sociodemographic and clinical characteristics (N=121)

	**Total(N=121)**	**Historical Control (n=97)**	**INSPIRE(n=24)**	**Cramer’s V**
Age, median (Q1-Q3)	70 (65 - 76)	70 (65 - 75)	70 (65 - 78)	d=0.16
<= 70	70 (57.9%)	58 (59.8%)	12 (50.0%)	0.08
>70	51 (42.1%)	39 (40.2%)	12 (50.0%)	
Sex				0.05
Male	67 (55.4%)	55 (56.7%)	12 (50.0%)	
Female	54 (44.6%)	42 (43.3%)	12 (50.0%)	
Race				0.15
White	87 (71.9%)	73 (75.3%)	14 (58.3%)	
Non-White	34 (28.1%)	24 (24.7%)	10 (41.7%)	
Frailty Score, median (Q1-Q3)	1 (0 - 2)	1 (0 - 2)	1 (0 - 2)	d=0.29
0 (frail)	40 (33.1%)	30 (30.9%)	10 (41.7%)	0.11
1 (pre-frail)	35 (28.9%)	30 (30.9%)	5 (20.8%)	
2 (robust)	46 (38.0%)	37 (38.1%)	9 (37.5%)	
Medical Comorbidities, median (Q1-Q3)	2 (1 - 4)	2 (2 - 4)	2 (0.5 - 4)	0.13
0	14 (11.6%)	8 (8.2%)	6 (25.0%)	0.22
1	17 (14.0%)	14 (14.4%)	3 (12.5%)	
2	31 (25.6%)	27 (27.8%)	4 (16.7%)	
3+	59 (48.8%)	48 (49.5%)	11 (45.8%)	
Resectability				0.14
Resectable	45 (37.2%)	34 (35.1%)	11 (47.8%)	
Borderline Resectable	24 (19.8%)	18 (18.6%)	6 (25.0%)	
Locally Advanced/Metastatic	52 (43.0%)	45 (46.4%)	7 (29.2%)	

### Outcomes

#### Treatment consistency with national guidelines

 Initial therapy was consistent with NCCN preferred treatment based on fitness in 63% of patients receiving INSPIRE intervention compared to 60% in the historical control group (V = 0.02; Table 2). Amongst patients receiving non-NCCN preferred treatment regimens, higher treatment intensity was more common than lower treatment intensity in both the INSPIRE intervention (89% vs. 11%) as well as historical controls (72% vs. 28%) (V = 0.15; Table 2).

**Table 2.** Comparison of clinical outcomes for INSPIRE intervention and historical control patients (N=121)

Outcome	Total (*n* = 121)	Historical Control (*n* = 97)	INSPIRE (*N* = 24)	Cramer’s V
Initial Dose Reduction	54 (44.6%)	46 (47.4%)	8 (33.3%)	0.11
Treatment modification	79 (65.3%)	66 (68.0%)	13 (54.2%)	0.12
Dose Reduction	41 (33.9%)	34 (35.1%)	7 (29.2%)	0.05
Dose Delay	70 (57.9%)	58 (59.8%)	12 (50.0%)	0.08
ED Visit	43 (35.5%)	33 (34.0%)	10 (41.7%)	0.06
Hospital Admission	56 (46.3%)	48 (49.5%)	8 (33.3%)	0.13
NCCN Treatment Consistency	73 (60.3%)	58 (59.8%)	15 (62.5%)	0.02
NCCN Treatment Inconsistency	48 (39.7%)	39 (40.2%)	9 (37.5%)	
Increased Intensity	36 (75.0%*)	28 (71.8%*)	8 (88.9%*)	0.15*
Decreased Intensity	12 (25.0%*)	11 (28.2%*)	1(11.1%*)	

* Percentage and Cramer’s V are calculated among patients with NCCN treatment discordance

#### Unplanned treatment modifications

**Fig. 2:**
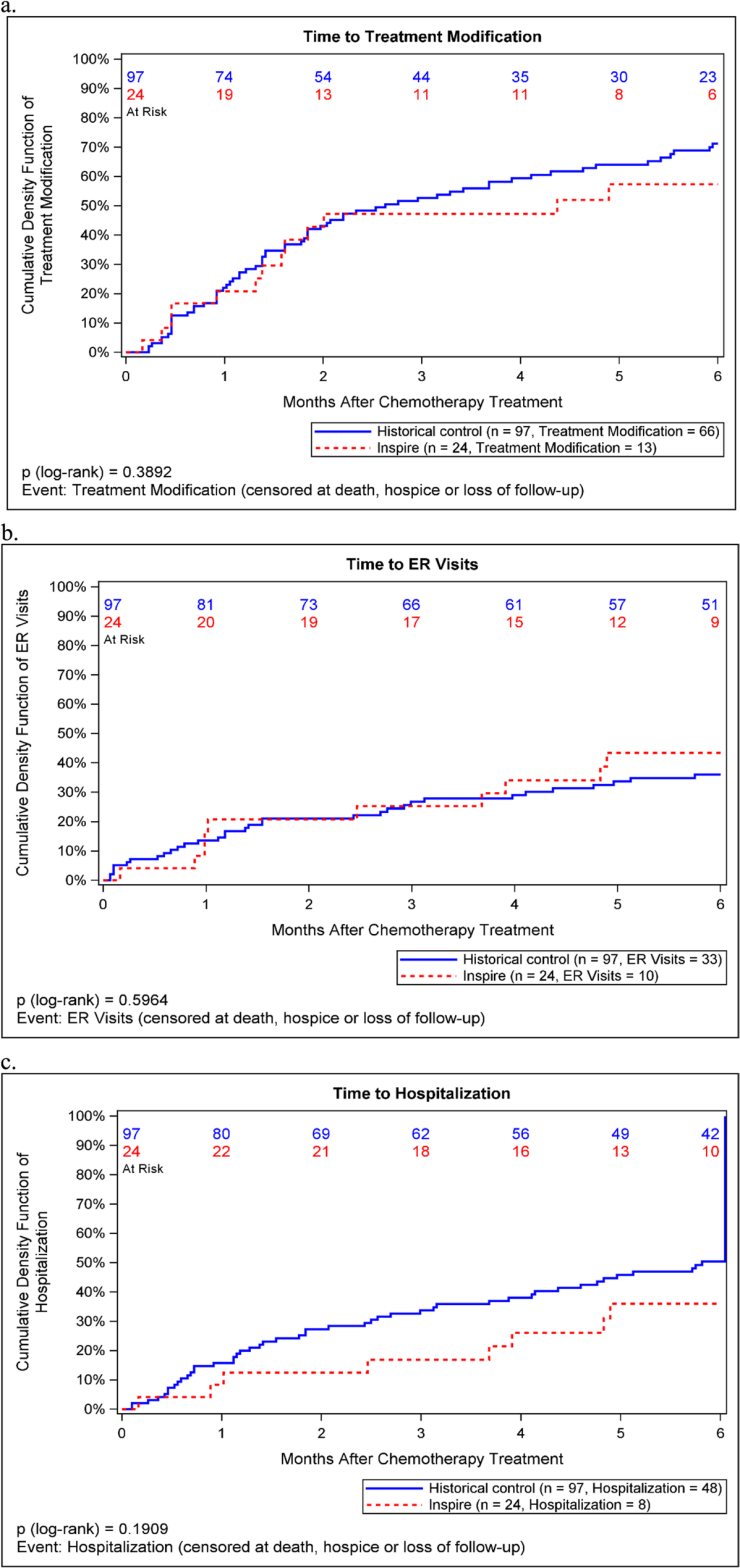
Cumulative incidence curves comparing INSPIRE intervention patients to historical controls for (**a**) time to treatment modification, (**b**) time to ER visit, and (**c**) time to hospitalization (N=121)

Within the first six months since treatment initiation, 68% of patients in the historical control cohort had unplanned treatment modifications compared to 54% in the INSPIRE intervention (V = 0.12). Compared to historical controls, patients receiving the INSPIRE intervention less often had dose reductions (29% vs. 35%, V = 0.05) or dose delays (50% vs. 60%, V = 0.08; Table 2). There was also no significant difference in the time to treatment modification comparing INSPIRE and historical control patients (*p* = 0.39, Fig. 2a).

#### Healthcare utilization

 The effects of the INSPIRE intervention on healthcare utilization were mixed. While patients receiving the INSPIRE intervention more often visited the ED within six months of treatment compared with historical controls (42% vs. 34%, V = 0.06), they were less often admitted to the hospital (33% vs. 50%, V = 0.13; Table 2). There was no significant difference in the time to ED visits (*p* = 0.60) or hospitalizations (*p* = 0.19) comparing INSPIRE and historical control patients (Figs. 2bc).

## Discussion

This study suggests that inclusion of GA fitness scores and patient preferences within MDTB discussions may increase the frequency of treatment consistency with national guidelines based on fitness and decrease frequencies of chemotherapy dose modifications and hospital admissions. To date, a growing body of literature indicates that successful completion and utilization of the GA results in multiple positive outcomes for patients with cancer. Mohile and colleagues have noted that completion of the GA helped to increase the number and quality of discussions regarding age-related concerns and preferences for cancer treatment [29]. Kenis and colleagues have noted that multiple different components of the GA provide accurate prognostic information regarding overall survival in older patients with cancer [30]. Our findings add to this literature by suggesting that successfully identifying patient fitness levels and treatment preferences via completion of the GA can yield beneficial effects.

While the GA provides an accurate assessment of patient fitness and treatment preferences, incorporating this information into the medical decision-making process in a standardized and efficient manner has remained a barrier to increased utilization in clinical practice. Through the INSPIRE intervention, we have shown that utilizing MDTBs as a platform to present GA data is both feasible and has promising effects [23]. MDTBs are utilized throughout the country to coordinate care for patients with cancer across various clinical team members. Several studies have reported on the benefit this coordinated care can provide to patients including MacDermid et al. who noted that being discussed at MDTBs was associated with an improvement in overall survival [31]. However, as noted by Rocque and colleagues, many MDTBs lack an efficient and effective method of incorporating the patient’s voice into these management decisions [32]. By employing the methods utilized in the INSPIRE intervention, we hope this will enable more MDTBs to incorporate patient-reported data into their decision-making process.

Distinct from many cancers, the NCCN guidelines on PDAC formally incorporate performance status (or patient fitness) into the recommended treatment algorithm [26]. Our findings suggest that the INSPIRE intervention may positively influence treatment consistency with national guidelines, as evidenced by higher rates of patients receiving NCCN-preferred regimens in the intervention group, which may result in more favorable downstream outcomes. Several studies in the PDAC literature, including those by Patel and colleagues and Khalaf and colleagues have documented associations between both unplanned chemotherapy modifications (dose reductions and/or dose delays) and increased healthcare utilization (ED visits and hospital admissions) with inferior clinical outcomes [33, 34]. Furthermore, studies such as those conducted by Mori and colleagues and Aziz and colleagues have noted that patients receiving high intensity treatment regimens are more likely to undergo unplanned chemotherapy modifications and are also more likely to present to the ED and be admitted to the hospital [35, 36]. These findings emphasize the importance of the decision-making process that determines a patient’s initial treatment regimen. The INSPIRE intervention intended to outline a method designed to better match each individual patient with their optimal treatment regimen through utilization of GA data during MDTB discussions. The data from this secondary analysis suggest an association between the INSPIRE intervention and decreased chemotherapy modifications and healthcare utilization. While not conclusive, this finding may be related to improved tailoring of individuals to appropriate treatment regimens.

It is important to note that our definition of consistency with national guidelines strictly correlated with patients receiving NCCN-preferred regimens and did not include patients receiving NCCN-other recommended regimens. This distinction likely contributed to the lower-than-expected consistency rates observed in both the intervention and control groups. Given the complexity of treatment decision-making in pancreatic ductal adenocarcinoma, factors such as comorbid conditions, overall health status, and individualized patient preferences may drive providers to select NCCN-other recommended regimens over NCCN-preferred regimens, even when the latter align more closely with guideline recommendations. Furthermore, there are scenarios in which a non–preferred regimen may be more appropriate for a given patient—for example, when molecular profiling identifies an actionable biomarker and the patient is eligible for targeted therapy. Additionally, NCCN guidelines base treatment recommendations on ECOG performance status, a metric shown to have both considerable inter-clinician variability as well as patient-physician variability [9–11]. Our study employed frailty scores derived from the GA, which were aligned into “ECOG equivalents.” Consequently, it is possible that patients we deemed “pre-frail” or “frail,” corresponding to worse ECOG scores, may have been perceived by physicians not utilizing the GA as more robust and therefore been eligible for more intensive treatment regimens. This discrepancy could explain why the rates of NCCN-preferred regimens were lower than expected in our cohort and may be appropriate for this population. Given that the guidelines are based on physician-reported ECOG, further assessment of the relationship between patient-reported fitness and outcomes for treatment approaches with varying intensity are needed.

While the results of the INSPIRE intervention are promising, the study has several limitations worth noting. Most notably, the pilot study included a small sample size appropriate for a feasibility study, which lacks power for formal statistical analysis and is intended to be hypothesis-generating. Furthermore, the study was conducted at a single institution in a non-randomized fashion and only assessed patients with PDAC. These limitations appropriately promote questions regarding the generalizability of this data as well as the applicability of these findings to cancers other than PDAC. Additionally, there were notable differences in baseline characteristics between the INSPIRE intervention cohort and the historical control group, including a higher proportion of patients with resectable disease in the INSPIRE group as well as greater frailty rates. These imbalances—stemming in part from the retrospective nature of the control data and our decision to include all eligible control patients to reduce selection bias—make it challenging to fully isolate the impact of the intervention itself from underlying differences between the groups. We acknowledge this as a key limitation that complicates interpretation of the observed outcomes. Also, due to the small sample size of the INSPIRE pilot intervention, further stratified analysis of these patients according to treatment consistency status was not possible. As such, the findings should be interpreted cautiously and considered as exploratory, warranting further investigation in larger, more rigorously powered studies to confirm any observed trends.

## Conclusion

This pilot study of the INSPIRE intervention revealed how utilizing methods to better incorporate ePROs into clinical decision-making may improve treatment consistency with national guidelines as well as minimize unplanned treatment modifications and healthcare utilization following chemotherapy.

## Supplementary Information

Supplementary Material 1.

Supplementary Material 2.

## Data Availability

The datasets used and/or analyzed during the current study are available from the corresponding author on reasonable request.

## Abbreviations

PDAC: pancreatic ductal adenocarcinoma
ePRO: electronic patient–reported outcome
MDTB: multidisciplinary tumor board
NCCN: National Comprehensive Cancer Network
FOLFIRINOX: floururacil, irinotecan, and oxaliplatin
HRQOL: health–related quality of life
GA: Geriatric Assessment
GI: Gastrointestinal
ED: Emergency Department
UAB: University of Alabama at Birmingham
WeCARE+: Web–Enabled Cancer and Aging Resilience Evaluation
ECOG: Eastern Cooperative Oncology Group
