# Supplementary material for: Impact of electronic patient-reported outcomes (ePRO) presentation in pancreatic cancer tumor board discussions on cancer outcomes: the INSPIRE intervention
Source: BMC Cancer. 2025 Dec 30;26:182. doi: 10.1186/s12885-025-14847-w (PMC12870527; doi:10.1186/s12885-025-14847-w)
Supplement: Supplementary file 1 — Supplementary Material 1. [file 12885_2025_14847_MOESM1_ESM.docx]

**Appendix A: Cancer and Aging Resilience Evaluation (CARE Survey)**

1. How many times have you fallen in the last 6 months?

**2.** Does your health limit you in walking one block?  Not limited at all  Limited a little  Limited a lot

**3.** Can you get to places out of walking distance …

Without help (drive your own car, or travel alone on buses or taxis);

With some help (need someone to help you or go with you when traveling); or

Are you unable to travel unless emergency arrangements are made for specialized vehicle like an ambulance?

1. Can you go shopping for groceries or clothes (assuming you have transportation)

Without help (taking care of all shopping needs yourself, assuming you had transportation);

With some help (need someone to go with you on shopping trips); or

Are you completely unable to do any shopping?

1. Can you prepare your own meals …

Without help (plan and cook all meals yourself);

With some help (can prepare somethings but unable to cook full meals yourself);

Are you completely unable to prepare any meals?

1. Can you do your housework …

Without help (can clean floors, etc.);

With some help (can do light housework but need help with heavy work); or

Are you completely unable to do any housework?

1. Can you take your own medicines …

Without help (in the right doses at the right time);

With some help (able to take medicine if someone prepares if for you and/or reminds you); or

Are you completely unable to take your medicines?

1. Can you handle your own money …

Without help (write checks, pay bills, etc.);

With some help (manage day-to-day buying but need help with managing your checkbook and paying your bills); or

Are you completely unable to handle money?

1. Can you get in and out of bed …

Without any help or aids;

With some help (either from a person or with the aid of some device); or

Are you totally dependent on someone else to lift you?

1. Can you dress and undress yourself …

Without any help (able to pick out clothes, dress and undress yourself);

With some help; or

Are you completely unable to dress and undress yourself?

1. Can you take a bath or shower …

Without help;

With some help (need help getting in and out of the tub or need special attachments); or

Are you completely unable to bathe yourself?

| **1.** In general, would you say your health is: | | | | |  | | |  | |  | | |  | |  |
| --- | --- | --- | --- | --- | --- | --- | --- | --- | --- | --- | --- | --- | --- | --- | --- |
|  | | | | | Excellent | | | Very  good | | Good | | | Fair | | Poor |
| **2.** In general, would you say your quality of life is: | | | | |  | | |  | |  | | |  | |  |
|  | | | | | Excellent | | | Very  good | | Good | | | Fair | | Poor |
| **3.** In general, how would you rate your physical health? | | | | |  | | |  | |  | | |  | |  |
|  | | | | | Excellent | | | Very  good | | Good | | | Fair | | Poor |
| **4.** In general, how would you rate your mental health, including your mood and your ability to think? | | | | |  | | |  | |  | | |  | |  |
|  | | | | | Excellent | | | Very  good | | Good | | | Fair | | Poor |
| **5.** In general, how would you rate your satisfaction with your social activities and relationships? | | | | |  | | |  | |  | | |  | |  |
|  | | | | | Excellent | | | Very  good | | Good | | | Fair | | Poor |
| **6.** In general, please rate how well you carry out your usual social activities and roles. (This includes activities at home, at work and in your community, and responsibilities as a parent, child, spouse, employee, friend, etc.) | | | | |  | | |  | |  | | |  | |  |
|  | | | | | Excellent | | | Very  good | | Good | | | Fair | | Poor |
| **7.** To what extent are you able to carry out your everyday physical activities such as walking, climbing stairs, carrying groceries, or moving a chair? | | | | |  | | |  | |  | | |  | |  |
|  | | | | | Completely | | | Mostly | | Moderately | | | A little | | Not at All |
| **8. In the past 7 days**, how often have you been bothered by emotional problems such as feeling anxious, depressed or irritable? | | | | |  | | |  | |  | | |  | |  |
|  | | | | | Never | | | Rarely | | Sometimes | | | Often | | Always |
| **9.** **In the past 7 days**, how would you rate your fatigue on average? | | | | |  | | |  | |  | | |  | |  |
|  | | | | | None | | | Mild | | Moderate | | | Severe | | Very Severe |
|  | | | | |  | | |  | |  | | |  | |  |
|  | No Pain |  |  |  | |  |  | |  | |  |  | |  | Worst Imaginable Pain |
| **10.** **In the past 7 days**, how would you rate your pain on average | 0 | 1 | 2 | 3 | | 4 | 5 | | 6 | | 7 | 8 | | 9 | 10 |

**Nutrition:**

**1. Weight.**

I currently weight about ______ pounds, and I am about ____ feet and ____ inches tall

One month ago I weighed about ______ pounds

Six months ago I weighed about ______ pounds

During the past two weeks my weight has:  decreased  not changed  increased

**2. Food Intake.**

As compared to my normal intake, I would rate my food intake during the past month as:

unchanged

more than usual

less than usual

I am now taking:

*normal food* but less than normal amount

little solid food

only liquids

only nutritional supplements

very little of anything

only tube feedings or only nutrition by vein

**3. Symptoms.** I have had the following problems that have kept me from eating enough during the past two weeks (check all that apply):

no eating problems  vomiting

no appetite, just did not feel like eating  diarrhea

nausea  dry mouth

constipation  smells bother me

mouth sores  feel full quickly

things taste funny or have no taste  fatigue

problems swallowing  pain; where? _______

other _______ (examples: depression, money, or dental problems)

**4. Activities and Function.**

Over the past month, I would generally rate my activity as:

Normal activity with no limitations

Not your normal self, but able to be up and about with fairly normal activities

Not feeling up to most things, but in bed or chair less than half the day

Able to do little activity and spend most of the day in bed or chair

Pretty much bedridden, rarely out of bed

| **KINDS OF SUPPORT**  **Do you have...** | **None of the time** | **A little of the time** | **Some of the time** | **Most of the time** | **All of the time** |
| --- | --- | --- | --- | --- | --- |
| **1.** Someone to help if you were confined to bed | ○ | ○ | ○ | ○ | ○ |
| **2.** Someone to take you to the doctor if needed | ○ | ○ | ○ | ○ | ○ |
| **3.** Someone to prepare your meals if you are unable to do it yourself | ○ | ○ | ○ | ○ | ○ |
| **4.** Someone to help with daily chores if you were sick | ○ | ○ | ○ | ○ | ○ |
| **5.** Someone to have a good time with | ○ | ○ | ○ | ○ | ○ |
| **6.** Someone to turn to for suggestions about how to deal with a personal problem | ○ | ○ | ○ | ○ | ○ |
| **7.** Someone who understands your problems | ○ | ○ | ○ | ○ | ○ |
| **8.** Someone to love and make you feel wanted | ○ | ○ | ○ | ○ | ○ |

**In the past 7 days…**

**Never Rarely Sometimes Often Always**

I felt fearful……………..……………………..

I found it hard to focus on anything

other than my anxiety………................

My worries overwhelmed me……………….

I felt uneasy…………………………………..

**Never Rarely Sometimes Often Always**

I felt worthless………………………………..

I felt helpless…………………………………

I felt depressed………………………………

I felt hopeless………………………………..

**Never Rarely Sometimes Often Very often**

My thinking has been slow…………………

It has seemed like my brain was not

working as usual…………………………

I have had to work harder than usual to

keep track of what I was doing…………

I have had trouble shifting back and forth between different activities that require

thinking…………………………………….

**9.** How many medications do you take on a daily basis?

**10.** How many other medical problems do you have besides your cancer?

**11.** Have you been seen in the ER (Emergency Room) in the past year?

Yes  No  Don’t know / Not sure

**12.** Have you been hospitalized (spent at least one night in the hospital) in the past year?

Yes  No  Don’t know / Not sure

1. During the past 4 weeks, how much of the time has your physical health or emotional problems interfered with your social activities (like visiting friends, relatives, etc.)?

| All of the time | Most of the time | Some of the time | A little of the time | None of the time |
| --- | --- | --- | --- | --- |

1. Compared to others your age, are your social activities more or less limited because of your physical or emotional problems?

| Much more limited than others | Somewhat more limited than others | About the same as others | Somewhat less limited than others | Much less limited than others |
| --- | --- | --- | --- | --- |

**3.** How is your eyesight (with glasses or contacts)?

|  |  |  |  |  |
| --- | --- | --- | --- | --- |
| Excellent | Good | Fair | Poor | Totally Blind |

**4.** How is your hearing (with a hearing aid, if needed)?

|  |  |  |  |  |
| --- | --- | --- | --- | --- |
| Excellent | Good | Fair | Poor | Totally Deaf |

**5.** Do you have to pay for more medical care than you can afford?

|  |  |  |  |  |
| --- | --- | --- | --- | --- |
| Strongly Agree | Agree | Uncertain | Disagree | Strongly Disagree |

**Your Health:** Do you have any of the following illnesses **at the present time**? If you fill in "yes," please tell us how much the illness interferes with your activities:

**IF YOU HAVE THE ILLNESS,**

**how much does it interfere with your activities?**

| **Illness** | **No** | **Yes** |  | **Not at all** | **Somewhat** | **A Great Deal** |
| --- | --- | --- | --- | --- | --- | --- |
| 1. Other cancers or leukemia | ○ | ○ | → | ○ | ○ | ○ |
| 2. Arthritis or rheumatism | ○ | ○ | → | ○ | ○ | ○ |
| 3. Glaucoma | ○ | ○ | → | ○ | ○ | ○ |
| 4. Emphysema or chronic bronchitis | ○ | ○ | → | ○ | ○ | ○ |
| 5. High blood pressure | ○ | ○ | → | ○ | ○ | ○ |
| 6. Heart disease | ○ | ○ | → | ○ | ○ | ○ |
| 7. Circulation trouble in arms or legs | ○ | ○ | → | ○ | ○ | ○ |
| 8. Diabetes | ○ | ○ | → | ○ | ○ | ○ |
| 9. Stomach or intestinal disorders | ○ | ○ | → | ○ | ○ | ○ |
| 10. Osteoporosis | ○ | ○ | → | ○ | ○ | ○ |
| 11. Chronic liver or kidney disease | ○ | ○ | → | ○ | ○ | ○ |
| 12. Stroke | ○ | ○ | → | ○ | ○ | ○ |
| 13. Depression | ○ | ○ | → | ○ | ○ | ○ |

**Demographics.**

**1.** What is the highest grade you finished in school?

| 1-8 grades  9-11 grades  High school graduate  Some college | Junior college degree  College degree (B.A./B.S.)  Some post-college work  Advanced degree |
| --- | --- |

**2.** What is your current marital status?

Single, never married  Divorced

Married  Widowed

Separated

**3.** What is your race? (check all that apply)

| White  Black or African American  Native Indian or Alaskan Native  Prefer not to answer | Asian  Native Hawaiian or Other Pacific Islander  Unknown |
| --- | --- |

**4.** What is your ethnicity?

Hispanic or Latino  Unknown

Non-Hispanic  Prefer not to answer

**5.** What is your current employment status? (check all that apply)

| Employed more than 32 hours per week  Employed less than 32 hours per week  Full-time or Part-time student  Homemaker  On medical leave | Disabled  Unemployed  Retired  Other: |
| --- | --- |

**6.** With whom do you live? (check all that apply)

| Spouse/Partner  Parent(s)/Parent(s)-In-Law  Live alone | Children aged 18 years or younger  Children aged 19 years or older  Other, specify: |
| --- | --- |

**THANK YOU for taking the time to complete this questionnaire!**

**Appendix B: Preference Survey**

**Patient Preferences**

Treatment for cancer can impact many aspects of a person’s life. We are interested in what are the most important things to consider when choosing a treatment. Please chose up to **three** of your biggest concerns:

☐ Physical side effects (ex. hair loss, neuropathy, nausea, fatigue)

☐ Emotional or mental side effects (ex. sadness, anxiety, memory)

☐ Ability to work

☐ Survival (ex. how long I have to live)

☐ Personal responsibilities (ex. caregiving, household responsibilities)

☐ Logistics and Convenience (ex. transportation, lodging, length of time in clinic, taking a pill vs. intravenous medicine, need to set up appointments, calls with insurance company)

☐ Out-of-pocket expenses (ex. co-pays, gas/parking money)

☐ Impact on activities of daily life (ex. bathing, dressing, nutrition, hobbies, exercise)

☐ Burden on family, friends, or care partners

☐ Important events or “bucket list” (ex. attend wedding, travel)

☐ Ability to be on a clinical trial or newly approved medication

☐ Sexual Concerns (ex. sexual intimacy, physical appearance)

☐ Fertility (ex. ability to have children)

Are the any other concerns that you have when making treatment decisions?

☐ Yes ☐ No If yes, what other concerns do you have? _______________________

**Next, we would like to ask you a few questions about common trade-offs in medical decisions.**

**Select which statement best represents how you feel about what’s important in life?**

☐ Quality of Line if all that matters

☐ Quality of Life is more important but Length of Life matters

☐ Length of Life is more important but Quality of Life matters

☐ Length of Life is all that matters

How important is quality of life to you?

☐ Not at all important

☐ Somewhat important

☐ Moderately important

☐ Quite important

☐ Very Important

How important is length of life to you?

☐ Not at all important

☐ Somewhat important

☐ Moderately important

☐ Quite important

☐ Very Important

**Appendix C: MDTB PRO Dashboard Example**
