# Supplementary material for: Impact of electronic patient-reported outcomes (ePRO) presentation in pancreatic cancer tumor board discussions on cancer outcomes: the INSPIRE intervention
Source: BMC Cancer. 2025 Dec 30;26:182. doi: 10.1186/s12885-025-14847-w (PMC12870527; doi:10.1186/s12885-025-14847-w)
Supplement: Supplementary file 2 — Supplementary Material 2. [file 12885_2025_14847_MOESM2_ESM.docx]

Supplementary Table 1. Sample sociodemographic and clinical characteristics of included and excluded patients (N=206).

|  | Total (n=206) | Excluded (N=85) | Included (n=121) | Cramer’s V |
| --- | --- | --- | --- | --- |
| Age, median (Q1-Q3) | 70 (65 - 76) | 70 (64 - 76) | 70 (65 - 76) | d=0.07 |
| <= 70 | 116 (56.3%) | 46 (54.1%) | 70 (57.9%) | 0.04 |
| > 70 | 90 (43.7%) | 39 (45.9%) | 51 (42.1%) |  |
| Sex |  |  |  | 0.07 |
| Male | 108 (52.4%) | 41 (48.2%) | 67 (55.4%) |  |
| Female | 98 (47.6%) | 44 (51.8%) | 54 (44.6%) |  |
| Race |  |  |  | 0.01 |
| White | 147 (71.4%) | 60 (70.6%) | 87 (71.9%) |  |
| Non-White | 59 (28.6%) | 25 (29.4%) | 34 (28.1%) |  |
| Frailty Score, median (Q1-Q3) | 1 (0 - 2) | 0 (0 - 1) | 1 (0 - 2) | d=0.44 |
| 0 (frail) | 86 (41.7%) | 46 (54.1%) | 40 (33.1%) | 0.22 |
| 1 (pre-frail) | 54 (26.2%) | 19 (22.4%) | 35 (28.9%) |  |
| 2 (robust) | 65 (31.6%) | 19 (22.4%) | 46 (38.0%) |  |
| Missing | 1 (0.5%) | 1 (1.2%) | 0 (0.0%) |  |
| Medical Comorbidities, median (Q1-Q3) | 3 (2 - 4) | 3 (2 - 4) | 2 (1 - 4) | d=0.23 |
| 0 | 22 (10.7%) | 8 (9.4%) | 14 (11.6%) | 0.13 |
| 1 | 28 (13.6%) | 11 (12.9%) | 17 (14.0%) |  |
| 2 | 45 (21.8%) | 14 (16.5%) | 31 (25.6%) |  |
| 3+ | 111 (53.9%) | 52 (61.2%) | 59 (48.8%) |  |
| Resectability |  |  |  | 0.15 |
| Resectable | 66 (32.0%) | 21 (24.7%) | 45 (37.2%) |  |
| Borderline Resectable | 35 (17.0%) | 11 (12.9%) | 24 (19.8%) |  |
| Locally Advanced/Metastatic | 97 (47.1%) | 45 (52.9%) | 52 (43.0%) |  |
| Unknown | 8 (3.9%) | 8 (9.4%) | 0 (0.0%) |  |
